# Influence of Milling Parameters on Mechanical Properties of AA7075 Aluminum under Corrosion Conditions

**DOI:** 10.3390/ma11091751

**Published:** 2018-09-17

**Authors:** María Jesús Martín, María José Cano, Germán Castillo, Manuel José Herrera, Francisco Martín

**Affiliations:** Civil, Material and Manufacturing Engineering Department, EII, University of Málaga, 29071 Málaga, Spain; mjcano@uma.es (M.J.C.); gcastillo@uma.es (G.C.); mherrera@uma.es (M.J.H.); fdmartin@uma.es (F.M.)

**Keywords:** machining, mechanical properties, corrosion, aluminum, flat specimen, AA7075

## Abstract

The paper describes an experimental study developed on the AA7075 T651, which is an aluminum alloy extensively used in the aeronautical industry. This work presents a double approach of investigation where there is no literature about previous research. This includes the analysis of the results obtained by the combination of mechanical and chemical actions on the mechanical properties of this material. On the one hand, the combinations of relevant milling parameters (feed rate, cutting speed) on flat samples (flat specimens have been selected by attempting to reproduce with the most accurate way the geometry and the type of machining process known as face milling is usually used in this manufacturing field). On the other hand, the stimulating effect of the corrosion by salt spray on selected batches of specimens was machined in the previous stage. Results from tensile tests performed on the whole of specimens allowed us to evaluate how the main mechanical properties (yield strength, tensile strength, and elongation at break) have been affected by the processes applied. Elongation at the break presents a reduction in an inverse order to feed a rate increase (up to 24.5%) and this reduction is extended (additional 19.17%) in specimens under corrosion conditions, which results in a greater fragility of the material.

## 1. Introduction

The aerospace industry requires materials with exceptional performance and long-term reliability. In this sense, aluminum and its alloys is one of the materials used most commonly in this field. This is thanks to some of its unique characteristics such as a high strength-to-weight, ease of fabrication, or its relative low cost. There are several different types of aluminum used but the 7075 Al alloy remains the baseline [1,2,3]. Part of this is due to its good balance of properties required for aerospace applications. Additionally, it is possible to apply sequences of mechanical and thermal treatments to produce annealed states as well as major combinations of characteristics through variations in treatment conditions. Taking into account that AA7075 T651 is widely used in aeronautics, this alloy will be the focus of this research. 

Nowadays, advanced composites consisting of a combination of high-strength stiff fibers embedded in a common matrix material [4] or titanium alloys are also widely being used in the aerospace industry. Both compete with aluminum [5,6] but present manufacturing difficulties especially in machining processes. Aluminum alloys can be machined rapidly and economically because of their micro-constituents that have important effects on machining characteristics [7,8,9]. In this way, numerous conventional machining processes can be performed on these materials (milling, drilling, and turning) by taking into account the different geometric characteristics required in each process [10]. Concretely, manufacturing of lightened plates of aluminum by face milling or profile milling is one of the most applied machining processes. Therefore, in this project, this type of machining process will be performed on the flat specimen to study in the attempt to reproduce as accurately as possible the way in which aircraft structures are made of. We studied the influence of different dominant parameters of this type of machining such as cutting speed, feed per tooth or cutting depth, fixing values of cutting speed and depth of cut, and taking the feed rate as the parameter to establish a degree of freedom in this experiment due to its significant influence on the roughness surface of the parts machined [11,12,13,14,15]. For this variable, a range of three values will be applied.

Traditionally, cutting fluids have been widely used in metalworking processes by providing cooling and lubrication while also preventing corrosion and facilitating the ejection of cut metal. However, their composition, usage, and disposal can negatively impact the environment and the health of exposed individuals by making necessary their reduction and even their elimination [16,17,18]. Specifically, in aluminum machining, the mixture of chip and cutting fluid make more difficult a correct recycling [19]. Other times, aluminum alloys are machined jointly with different types of materials such as composites or polymers reinforced with carbon fiber, which do not have a satisfactory performance with cutting fluids. Because of this, dry cutting is presented as an alternative to minimize or avoid the use of cutting fluid [20,21,22,23]. However, dry machining demands more rigorous requirements in machining operations and affects largely the surface finish [24]. 

Taking as a starting point the previous machining conditions, it is needed to define combinations of cutting parameter values to improve the mechanical behavior of the material studied [25]. This premise will define the first of the three stages of the present project. 

Studies about AA7075 alloy hardly exists in literature, which is remarkable when considering that this material is widely used in aeronautic components.

On the other hand, it is widely known that corrosion is a universal enemy whose presence is inevitable in a productive process or any other. Corrosion can be defined as the chemical or electrochemical reaction of a metal or alloy with its surrounding environment and with the consequent deterioration of its properties [26,27]. One of the most obvious manifestations of corrosion is its direct effect on the surface of the metal subject. This allows us to establish that this relationship provokes a stimulant effect on the surface geometric imposed [28] and, therefore, has a greater influence on the variation of the mechanical properties of the material subjected to a corrosive environment.

It is possible to distinguish between corrosion by oxidation of the metal with the formation of metal cations and the dissolution of a metal in other metals or molten salts. There is a form of corrosion in which two effects are superimposed: the first one is chemical or electrochemical, which constitutes the corrosion itself, and the other one is mechanical. For example, the process of corrosion-erosion or corrosion under tension. The basic corrosion reaction is defined by the transit of the metal or alloy from its elementary form to the ionic or combined form (Equation (1)).

Me → Me^n+^ + ne^−^ (1)

n being the number of free electrons.

To complete the process, electrons must be fixed by affinity (an oxidant) by some substances that are present in the medium in contact such as oxygen. The elemental metal through the corrosion mechanism returns to the combined form by making oxides, sulfides, and more including the state in which metals are usually found in nature and are thermodynamically more stable.

It is possible to classify corrosion processes using different criteria. For example, considering the morphology of the attack, it could be described as uniform or localized (selective corrosion, intergranular attacks, among others) [29,30]. However, the most interesting scientific classification is based on the mechanism that produces this phenomenon. From this point of view, corrosion will be produced electrochemically by electrochemical battery cells on the metallic surface and, therefore, does not produce the same effect on the whole metallic surface due to the cathodic regions not being attacked. This type of corrosion appears when metal materials are in contact with electrolytic conductivity media specifically with water, salt solutions, or the simple humidity of the atmosphere and soils. The other fundamental type of corrosion occurs in conditions of absence of humidity on the complete metal surface at high temperatures. 

The phenomenon of stress corrosion cracking is typical of alloys including passive ones. This phenomenon is characterized by the appearance of cracks that advance in an approximately normal direction with respect to the application of tension [31,32]. Stress corrosion cracking results from the conjoint action of three components.

(a) Material under tensions higher than a defined threshold, whether applied or residual.
(b) Material susceptible to cracking, determined by the composition of the alloy, its microstructure or its grain size.
(c) Material in contact with a specific aggressive environment for it.

Regarding the first condition, it is important to emphasize that tension cannot only be applied when the alloy is part of a structural element but can be a residual tension arising from its forming process or its thermal history.

Previously described in this project, electrochemical corrosion under wet and saline environment has mainly been reproduced. Nevertheless, it also is possible to consider the stress corrosion cracking phenomenon, which could be implicated because of the notch effect. In our study, the notch effect in the sample will depend on the different levels of machining obtained by means of the variation of the milling parameters.

Machining conditions imposed on specimen batches and the corrosive environment subsequently applied to them allow us to analyze the influence of the ones on the mechanical properties of the material studied. Sometimes corrosion and machining working jointly can cause weakness and fragility of the material, which are able to result in a reduction of its tensile strength [33,34] and yield strength as well as a decrease in its percentage of elongation. 

## 2. Materials and Methods 

Because of the high interest within the aeronautic scope in the use of light alloys especially aluminum ones, this work is focused on the mechanical behavior analysis of one of the most important alloys used in this field including the AA7075 T651 alloy (thermal treatment and tensile controlled of 1.5% to 3% (according to the manufacturer). It starts from the aluminum sheet 8 mm thick, EN AW7075 (AlZn5, 5MgCu) (Table 1). The choice of this thickness is conditioned by the fact that both faces of the sheet are going to be machined under different cutting parameters by following the purpose of doing the analysis of the influence of these machining conditions on samples subject to a corrosive environment. Vibration effects from the machine operation on the work held to the table can produce warps and waves. This situation obliges it to choose a particular initial thickness, according to the last 4 mm thick. Samples are created by the top face and edge machining. Because the main goal of this work is the study of the influence of the milling parameters on the corrosion effect on the samples and the effect on their mechanical behavior, it is absolutely necessary to perform a controlled material-removal process [8,12]. 

It has been used with the three axes milling machine Kondia K600 with CNC control FAGOR 8050. The rotating face milling cutter is a face mill STD B0 0302 of 63 mm external diameter with a major cutting edge angle (or Entering angle) of 90° that holds six inserts STD ISO APKT 160408 B0 0138 of K10/20 quality. The rotating edge milling cutter is an edge mill STD FP07 of ASP material (high performance steel from the powder metallurgy process) and cutting length of 19 mm and an 8 mm external diameter with 4 teeth or cutting edges.

**Figure 1:**
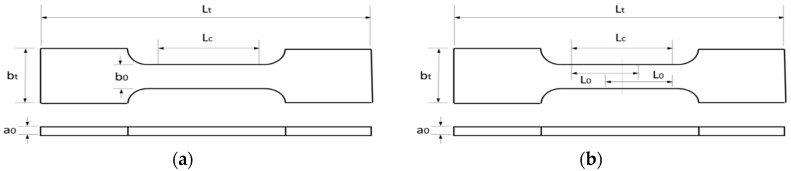
(**a**) Tensile specimen dimension; (**b**) Gage section and measurement distances.

Due to the requirements in standard UNE-EN ISO 6892-1:2017 (ISO 6892-1:2016) [35], which regulates tensile tests, it has been considered the typical tensile flat specimen shown in Figure 1.

Flat specimens will be used with dimensions as specified below by considering a proportional flat specimen.

*a*_0_ = 4 mm, *b*_0_ = 20 mm, $S_{0}=a_{0}\cdot b_{0}$
*S*_0_ = original cross sectional area
*L*_0_ = original gauge length
$L_{0}=K\cdot \;\sqrt{S_{0}}$, K = 5.65
*L*_0_ = 50 mm
*L_c_* = paralell length
$L_{c}\geq \;L_{o}+1.5\cdot \;\sqrt{S_{0}}$
*L_c_* = 80 mm
*L_t_* = total length

Transition radius of 12 mm are defined between gauge and gripping sections.

This study analyzes the influence of machining parameters on the specimens subjected to corrosion conditions. Milling machining has been selected as the machining operation to be carried out because it is said to be an essential process in the aeronautical industry. The next step consists of establishing the machining conditions that will be applied. The three primary input control parameters that will be taken into account are: cutting speed (*Vc*), depth of cut (*a_p_*), and feed rate (*F*). The first two variable values will be in accordance with the cutter tool geometry and they will remain constant. The feed rate (*F*) will be the parameter to be modified. Three values chosen from the values range provided by the manufacturer will be given. Consequently, three different study batches of specimens will be generated. Following the supplier recommendations and the milling tool characteristics, the depth of cut must be less than 7 mm in face milling and less than 19 mm in profile or edge milling. In this experience, it is established that there will be 2 mm of the depth of cut in the face milling and 4 mm in the other. Referring to cutting speed (*Vc*), due to the milling tools selected, the reference interval will be a 200 to 700 m/mm range for face milling and a 100 to 150 m/mm range for edge milling (manufacturer’s information). Then, by taking the lowest value of cutting speed from each range, the spindle speed is determined (Equation (2)).

(2) $$\;s\;\left(\frac{\mathrm{rev}}{\min}\right)=\;\frac{Vc\;\left(\frac{m}{\min}\right)\cdot 1000\;\left(\frac{\mathrm{mm}}{m}\right)}{\pi \;\cdot \;D\;\left(\mathrm{mm}\right)}\;$$

Spindle speed in face milling (Equation (3)).

(3) $$\;s=\;\frac{200\cdot 1000}{\pi \;\cdot \;63}\;=1010.50\;$$

Spindle speed in edge milling (Equation (4)).

(4) $$\;s=\;\frac{100\cdot 1000}{\pi \;\cdot \;8}=3978.86\;$$

where

*s* = revolution per minute of the cutter,
*Vc* = lineal cutting speed of the material in m/mm,
*D* = diameter of the cutter in mm

Feed rate comes from the next equation (Equation (5)).

(5) $$\;F\left(\frac{\mathrm{mm}}{\min}\right)=s\left(\frac{\mathrm{rev}}{\min}\right)\cdot Z\;\left(\mathrm{teeth}\right)\cdot fz\left(\frac{\mathrm{mm}}{\mathrm{tooth}}\right)\;$$

with

*fz* = movement per tooth of cutter in mm
*Z* = number of teeth of cutter

For feed per tooth, manufacturer recommendations indicate a range of 0.1 to 0.31 mm using the face milling tool. Experiments will be conducted taking three feed per tooth levels: 0.1 mm, 0.2 mm, and 0.3 mm. Therefore, considering these values on face machining, *F*_1*F*_, *F*_2*F*_*,* and *F*_3*F*_, identify the feed rate established for batch 1, batch 2, and batch 3, respectively (Equations (6)–(8)).

(6) $$\;F_{1F}=1010.50\cdot 6\cdot 0.1\;=606.30\;$$

(7) $$\;F_{2F}=1010.50\cdot 6\cdot 0.2\;=1212.60\;$$

(8) $$\;F_{3F}=1010.50\cdot 6\cdot 0.3\;=1818.90\;$$

By taking the previous three feed rate levels on profile or edge machining, *F_1E_*, *F_2E_,* and *F_3E_* are defined below (Equations (9)–(11)).

(9) $$\;F_{1E}=3978.86\cdot 4\cdot 0.06\;=954.93\;$$

(10) $$\;F_{2E}=3978.86\cdot 4\cdot 0.065\;=1034.50\;$$

(11) $$\;F_{3E}=3978.86\cdot 4\cdot 0.07\;=1114.08\;$$

Machining conditions are included in Table 2.

**Figure 2:**
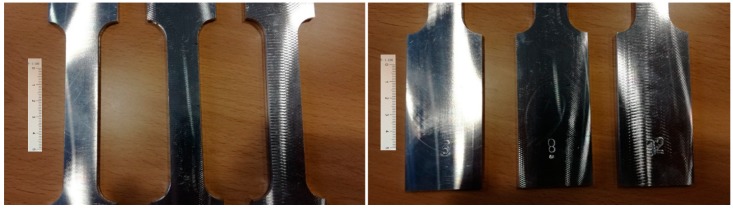
Machined samples from batches 1, 2, and 3 (scale bar in mm).

Figure 2 shows different finish surface levels depending on the cutting conditions, which includes sample numbers 3, 8, and 12 from the batches 1, 2, and 3, respectively.

Once specimens have been machined under the cutting conditions defined in Table 2 from each batch, one sample is defined as a “control sample” and the three others will be subjected to a salt spray testing. It is widely used as a rapid method for evaluating material performance under highly corrosive conditions.

The aim of this experience is the evaluation of the influence of cutting parameters by using corrosion as an enhancer medium on the mechanical behavior of aluminum alloys AA7075 T651.

Previously, the application of the corrosion test, the thickness, and width of each of the specimens was measured at different points of the gauge length by calculating the average section of each specimen in that zone.

Afterward, samples are exposed to salt fog or spray that is even distributed among the samples inside a testing chamber. The salt spray (salt fog) chamber consists of a fog chamber, which is a salt solution reservoir, a supply of suitably conditioned compressed air, one or more atomizing nozzles, specimen supports, provision for heating the chamber, and necessary means of control. In this case, the apparatus is HSN400 Heraeus Votsch. Samples have been suspended between 15° and 30° from the vertical parallel to the principal direction of the flow of fog.

This practice provides a controlled corrosive environment by chloride solution to produce relative corrosion in the specimens exposed in the test chamber. The salt fog testing conditions applied are:

- The salt solution is a solution of sodium chloride dissolved in distilled water with (50 ± 5) g/L concentration
- The exposure zone of the salt spray chamber has been maintained at 35 °C ± 2 °C
- The pH of the salt solution is such that, when atomized to 35 °C, the collected solution will be in a pH range between 6.5 to 7.2. The registered pH values have approached 6.5 and were measured by pH test paper.
- Spraying has been controlled by the average collected speed of atomized solution in minimum periods of 24 h. The spraying range in a horizontal manifold of 80 cm^2^ must be 1 to 2.5 mL/h. The volumes collected every 24 h have come close to 50 mL. Lastly, the period of exposure reached 168 h.

At the end of the test, specimens have been carefully removed and dipped in distilled water to remove salt and deposits from their surface and then they are immediately dried. 

**Figure 3:**
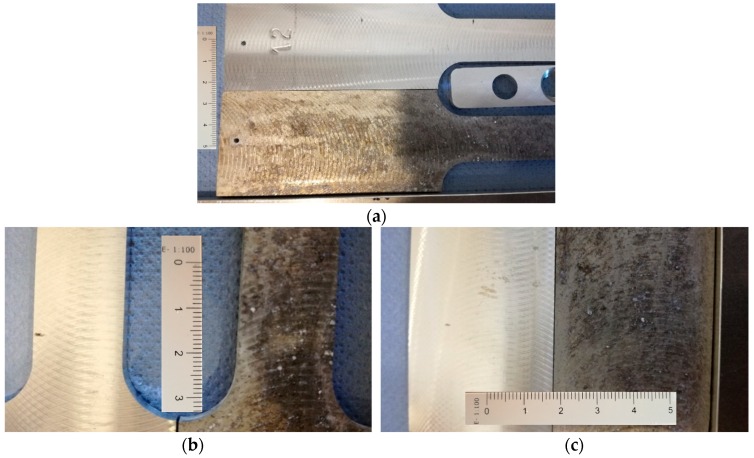
(**a**–**c**) Subject and no subject to corrosion specimens (scale bar in mm).

Once dried, they are wrapped in cellulose with desiccants. They will be kept in a watertight compartment until the next step. Figure 3a–c show the effect of the corrosive process on the samples tested.

Along with finished previous stages, the machined process, and the salt spray test, specimens will be subjected to tensile tests. They will provide information about the strength or their mechanical behavior.

The equipment used is a universal testing machine for tensile/compression/flexure 1 to 500 kN, ME 405 SERVOSIS, controlled by PCD2K test software (version 1.2) [36] that will give a test report with the required calculations and test results, data, and graphics. This testing machine works with two load cells of which the 100 kN one will be used in this experiment. 

**Figure 4:**
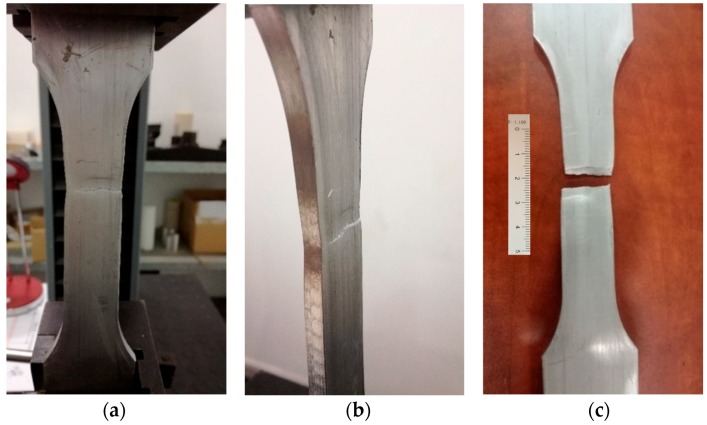
(**a**,**b**) Sample during the test; (**c**) Sample after the test (scale bar in mm).

Tensile tests are performed at a room temperature range between 10 °C and 35 °C (Figure 4). The sample dimensions have been described before. They are proportional with gage length, rectangular cross-sectional area, and soft transition radius connecting gage and gripping sections. Elongation at the break will be evaluated by measuring the length increase between the initial marks of the samples. The displacement speed of wedge grips is fixed in 1 mm/min [35].

The tensile force is recorded as a function of the increase in gage length. Such plots of the tensile force versus tensile elongation would be normalized to the Stress-Strain curve.

- Engineering stress: $\sigma =\;\frac{F}{S_{0}}\;$ where *F* is the tensile force and *S*_0_ is the initial cross-sectional area of the gage section.
- Nominal engineering strain: $\varepsilon_{n}\;=\;\frac{\left(L_{u}-\;L_{0}\right)}{L_{0}}\;$ where *L*_0_ is the initial gage length and *L_u_* is the final one.

**Figure 5:**
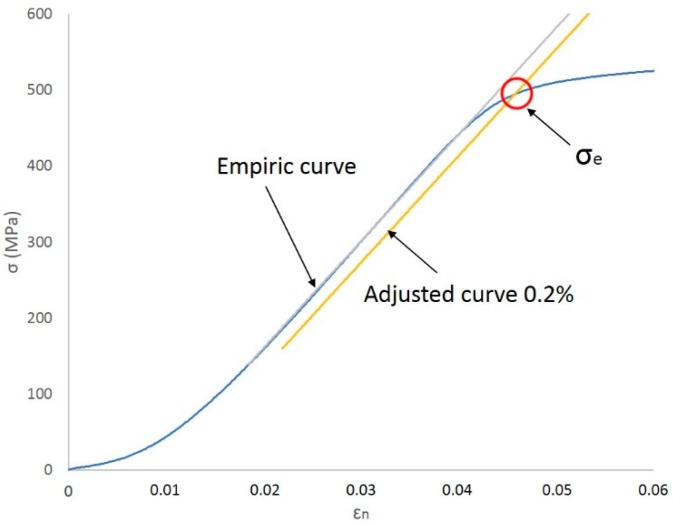
Elastic limit in an adjusted curve.

Taking this curve as a starting point, the value of the conventional elastic limit is determined by applying a linear regression method to the registers of the proportional elastic zone of the curve. Due to the need of a high goodness of fit of the data to the mathematical relationship, the correlation coefficient, *R*^2^, will not be less than 0.999. Once this curve has been adjusted again to the estimated deformation of 0.2%, the cut point of this new curve with the initial curve adjusted will provide the value of the elastic limit corresponding to the material tested (*σ_e_*) (Figure 5). Last instant of the tensile test is delimited by tensile strength *σ_R_*. 

Regarding the elongation at break, *A*, it will be calculated from the next expression.

$$\;A=\;\frac{L_{u}-\;L_{0}}{L_{0}}\cdot 100\;$$

with *L*_0_ = initial length and *L_u_* = length after a break. Measuring *L_u_* is especially critical because it requires a careful approach to ensure the best contact between both parts. This distance has been measured using a digital Vernier caliper of precision 0.01 mm.

## 3. Results and Discussion

Once the tensile tests have been done on both types of flat specimens considered, the denominated “control samples” and ones that have been previously subjected to a corrosive environment (specifically identified with the letter “C”) as well as the parameter values that characterize their mechanical behavior, yield strength, tensile strength, and elongation are analyzed. 

Table 3 presents the set of results obtained.

From these results, it is possible to do comparative evaluations between the controlled parameters on each specimen by considering the different variables on influence.

- Kind of specimen
- Corrosive action
- Milling machining conditions

**Figure 6:**
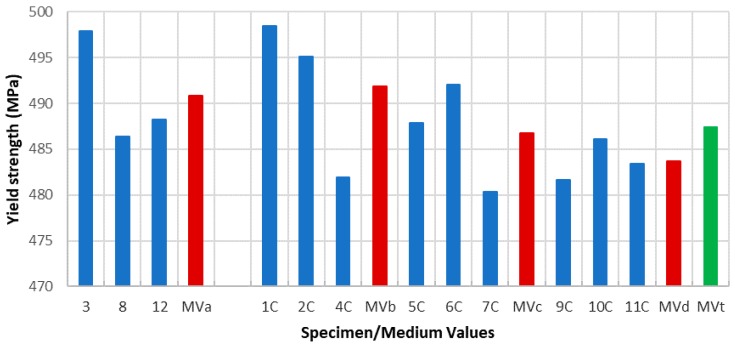
Yield strength vs. specimen and medium values.

**Figure 7:**
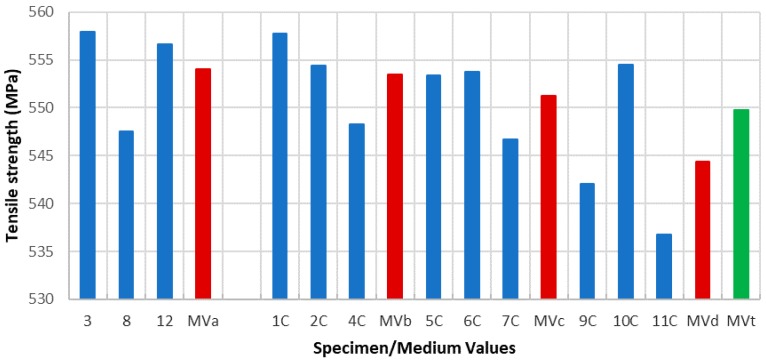
Tensile strength vs. specimen and medium values.

**Figure 8:**
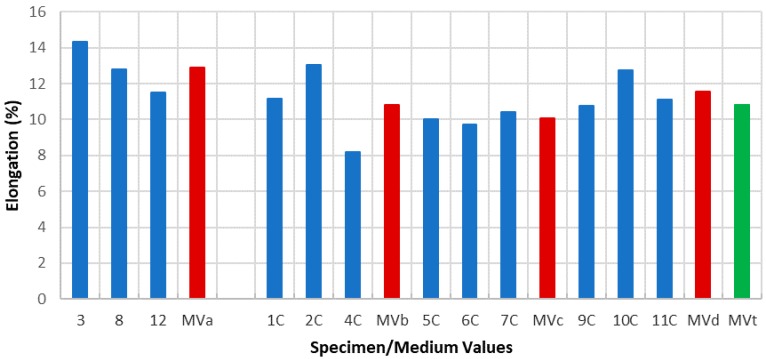
Elongation vs. specimen and medium values.

**Figure 9:**
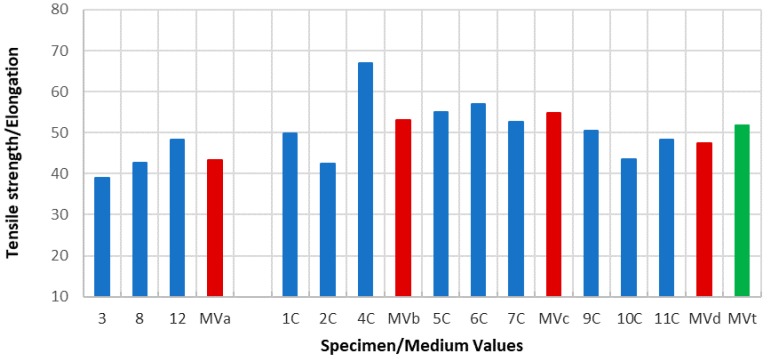
Tensile strength/elongation relation values and medium values.

Due to the specimens’ dimensions, it is necessary to calculate not only the absolute values of the mechanical parameters (elastic limit, tensile strength, and elongation at break) but the relative values of these three parameters, according to each specimen cross section (*S*_0_). Thus, the next tables and graphics show results from the evaluation of yield strength and yield strength/section relation (Table 4, Figure 6), tensile strength and tensile strength/section relation (Table 5, Figure 7), elongation to break and elongation at break/section relation (Table 6, Figure 8), and relation tensile strength/elongation at break (Table 7, Figure 9).

Data shown in Table 4, Table 5, Table 6 and Table 7 and Figure 6, Figure 7, Figure 8 and Figure 9 express those results relative to specimens not subjected to a corrosive environment (“control specimens”) and the three milling machined conditions (specimens 3, 8, and 12). In addition, medium values have been calculated and identified as MVa (medium value a). On the other side, Specimens 1C, 2C, and 4C belongs to batch 1 and they have been exposed to salt fog. Its corresponding medium value is MVb. Specimens from batch 2 including 5C, 6C, and 7C have suffered a corrosive attack. In this case, it is assigned the medium value MVc. The three next specimens include 9C, 10C, and 11C from batch 3 and under corrosive action present a medium value identified by MVd. Eventually, the last column shows the medium value of the three previous medium values from the batches of samples subjected to corrosion conditions that include MVb, MVc, and MVd. This parameter, MVt, represents the total medium value corresponding to the behavior of the studied variable.

**Figure 10:**
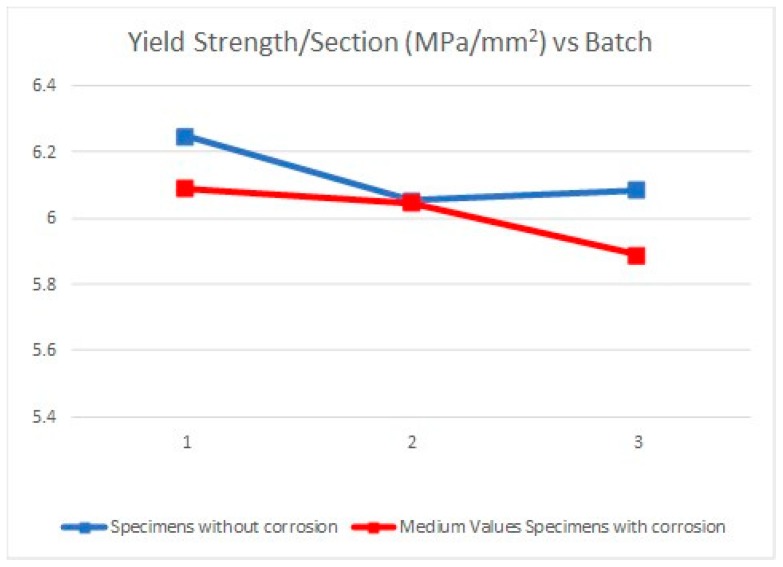
Yield strength/Section vs. Batch.

**Figure 11:**
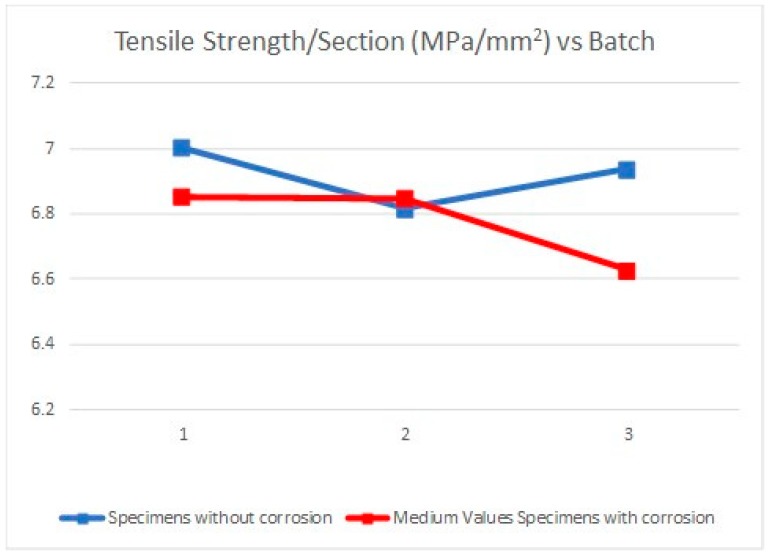
Tensile strength/Section vs. Batch.

Establishing relations between the samples areas and the machining parameters considered allows us to make a more accurate evaluation of how their mechanical behavior is affected by the machining conditions. According to standard deviations included in Table 4, Table 5, Table 6 and Table 7, the results show a reduced dispersion when cross sections are taken into account. Table 8 and Table 9 and Figure 10 and Figure 11 show the percentage variations of the elastic limit and the tensile strength. As shown, both percentages do not exceed the 4.6% between extreme values from each batch. Concretely, the reduced difference that both parameters present in batch 2 may indicate that, for this combination of machining parameters, the variation of the micro-geometry does not significantly affect the mechanical properties considered (tensile strength and elastic limit).

**Figure 12:**
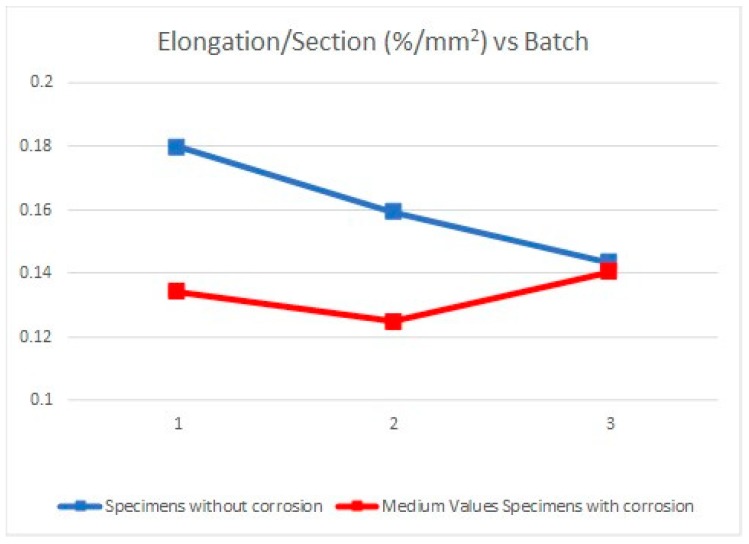
Elongation/Section vs. Batch.

In the opposite, the elongation at break is strongly influenced by the machining process and the activator effect of the corrosion on the surface roughness of each specimen (Table 10, Figure 12). In this sense, the increasing roughness level dependent on the machining conditions (*R_a_*_1_ *< R_a_*_2_ *< R_a_*_3_) prompts a fragility increase and consequently a lesser percentage elongation. Likewise, a similar situation is presented in specimens exposed to a corrosive environment and machined under cutting conditions identified as batch 1 and batch 2. In this case, not only is there a decrease on the percentage elongation but that decrease is very significant. Furthermore, an important difference appears with the percentage elongation of the “control specimen” being 33.97% in batch 1 and 27.60% in batch 2. These results show how the Aluminum 7075 ductility can be affected by the machining conditions applied.

However, batch 3 does not present the same behavior than batches described before. A similar comparison between values from the “control specimen” and specimens under corrosion conditions produces a result of 2.06%. The amount is especially insignificant compared to the results obtained with batches 1 and 2. Analyzing these results, it can be deduced that the combination of cutting depth, feed per teeth, and cutting speed in batch 3 reduces strongly the stimulator effect of the corrosion in relation to its fragility. These are not, nevertheless, the suitable machining conditions because the higher increase of the feed per tooth produces a rise in surface roughness and a fall in the percentage elongation.

Taking absolute values (without relation to cross sections), the conclusions deduced from the study of each one of the parameters contemplated in the different conditions of the test reveals that, in the case of the elastic limit, the similar behavior under different machining conditions and the average value obtained from the specimens without a corrosive action is 490.82 MPa. Therefore, we can establish this value as the elastic limit value of the material under study. For the samples subjected to a corrosive attack, the evolution of the average values of the yield strength is such that the result from batch 1, 491.85 MPa, is insignificant while it is demonstrated that a higher influence on the value of the elastic limit for the samples from batches 2 and 3. It can be observed as a downward trend of this evolution. The result is 486.74 MPa, which is the value of batch 2, and 483.72 MPa, which is the value for batch 3. This tendency is evident in the total average from the set of specimens under corrosion. This parameter (MVt) offers the value of 487.43 MPa.

Regarding the tensile strength, the evolution presents a similar aspect than the previous case. The average value of samples 3, 8, and 12 is 554.01 MPa while those one from the batches 1, 2, and 3 are 553.47 MPa, 551.27 MPa, and 554.42 MPa, respectively, which offers an average value of 549.72 MPa. It can be seen that a similar downward trend of the tensile strength occurs, according to the three different machined conditions.

Referring to elongation at break, results evidence a greater difference of the behavior of the samples subjected to corrosion tests. The summarized results according to the medium values obtained are: Average value of elongation at break for, without corrosion, 12.87% (similar to usual values in alloys of this type), specimens from corrosion tests and batch 1, 10.81%, batch 2, 10.05%, and batch 3, 11.54%, resulting in a total medium value of 10.80%. Overall, the decrease in the value of the percentage of elongation is even greater than in the two previous parameters contemplated.

Due to the tensile strength and elongation at break having been obtained by procedures of different empirical nature, mechanical testing in the first case and geometrical measurements in the second one establish the level of reliability offered by the two methods employed based on a relation that has been established between them with an eminently qualitative character. Since the reduction of the values of elongation is greater than that presented by the tensile strength values about the set of specimens subjected to corrosion, the medium values obtained for the considered relationship must be higher in the three series studied under these conditions (43.38 of MVa, against values of 53.12 of MVb, 54.88 of MVc, 47.40 of MVd and, therefore, 51.80 of MVt).

## 4. Conclusions

This work is supposed to present a new approach in the study of the response of the AA7075 T651 alloy, which is widely used in the aeronautic industry, to different mechanical and chemical actions such as milling machining and the corrosion process. In this sense, this work deals with a study field where it hardly exists in the literature.

Considering typical processes used in aeronautics, dry face milling has been selected as the machining operation to perform on the flat samples (different combinations by fixing cutting speed and depth of cut and modifying the feed rate). The corrosive environment has been obtained by means of a salt fog chamber. 

Different specific combinations of geometrical and technological cutting parameters are applied on flat specimens, which have been divided in two study groups. Samples belong to the second group and have been additionally exposed to a corrosive environment.

Based on the results obtained, it can be clearly seen as a relevant influence of both processes. On the one hand, whatever combination of milling parameters is applied, samples without a corrosion effect show an insignificant variation of their mechanical property values (2.37% in tensile strength and 1.90% in yield strength). However, the elongation at break presents a great reduction (24.5%) that varies inversely with the feed rate increase. Consequently, a higher feed rate results in a greater fragility of the material.

On the other hand, analyzing specimens machined and subjected to corrosion, results reveal that this phenomenon provokes a strong influence on the elongation at break. The elongation reduction follows a similar tendency with respect to samples without corrosion, but these samples present an evident greater fragility with an average elongation of 10.80% in comparison to the 12.87% of the first ones. Therefore, it produced an additional significant reduction of the 19.17%.

It is necessary to point out that all these observations are only valid within the tested range of milling parameters.

## Figures and Tables

**Table 1:** AA7075 alloy composition.

%	Si	Fe	Cu	Mn	Mg	Cr	Zn	Ti	Others Elements		Al
Minimum	-	-	1.20	-	2.10	0.18	5.10	-	Zr + Ti	Total	-
Maximum	0.40	0.50	2.00	0.30	2.90	0.28	6.10	0.20	0.25	0.15	rest

**Table 2:** Machining conditions.

**Batch**	**Face Milling *V_c_* (m/min)**	**Face Milling *a_p_* (mm)**	**Face Milling *F_F_* (mm/min)**	**Edge Milling *V_c_* (m/min)**	**Edge Milling *a_p_* (mm)**	**Edge Milling *F_E_* (mm/min)**
1	200	2	606.30	100	4	954.93
2	200	2	1212.60	100	4	1034.50
3	200	2	1818.90	100	4	1114.08

**Table 3:** Testing and measurement results.

Specimen	Section (mm^2^)	Yield Strength 0.2% *σ_e_* (MPa)	Tensile Strength *σ_R_* (MPa)	Elongation at Break *A* (%)
1C	80.80	498.50	557.75	11.18
2C	79.41	495.11	554.36	13.06
3	79.69	497.90	557.90	14.32
4C	82.16	481.94	548.30	8.18
5C	79.35	487.87	553.37	10.04
6C	80.72	492.02	553.73	9.72
7C	81.48	480.32	546.70	10.40
8	80.34	486.37	547.49	12.80
9C	82.76	481.65	542.09	10.74
10C	82.92	486.11	554.46	12.76
11C	80.81	483.39	536.72	11.12
12	80.24	488.20	556.63	11.50

**Table 4:** Yield strength and yield strength/section relation values per specimen and Medium values.

Specimen	Yield Strength	Yield Strength/Section
	(MPa)	(MPa/mm^2^)
3	497.90	6.25
8	486.37	6.05
12	488.20	6.08
Medium value a (3, 8, 12)	490.82	6.13
Standard deviation	6.20	0.11
1C	498.50	6.17
2C	495.11	6.23
4C	481.94	5.87
Medium value b (1C, 2C, 4C)	491.85	6.09
Standard deviation	8.75	0.19
5C	487.87	6.15
6C	492.02	6.09
7C	480.32	5.89
Medium value c (5C, 6C, 7C)	486.74	6.05
Standard deviation	5.93	0.14
9C	481.65	5.82
10C	486.11	5.86
11C	483.39	5.98
Medium value d (9C, 10C, 11C)	483.72	5.89
Standard deviation	2.25	0.08
Corrosion total medium value (MVt = (MVb + MVc + MVd)/3)	487.43	6.01
Standard deviation	4.11	0.11

**Table 5:** Tensile strength and tensile strength/section relation values per specimen and medium values.

Specimen	Tensile Strength	Tensile Strength/Section
	(MPa)	(MPa/mm^2^)
3	557.90	7.00
8	547.49	6.81
12	556.63	6.94
Medium Value a (3, 8, 12)	554.01	6.92
Standard deviation	5.68	0.10
1C	557.75	6.90
2C	554.36	6.98
4C	548.30	6.67
Medium value b (1C, 2C, 4C)	553.47	6.85
Standard deviation	4.79	0.16
5C	553.37	6.97
6C	553.73	6.86
7C	546.70	6.71
Medium value c (5C, 6C, 7C)	551.27	6.81
Standard deviation	3.96	0.13
9C	542.09	6.55
10C	554.46	6.67
11C	536.72	6.64
Medium value d (9C, 10C, 11C)	544.42	6.94
Standard deviation	9.10	0.06
Corrosion total medium value	549.72	6.77
(MVt = (MVb + MVc + MVd)/3)		
Standard deviation	4.72	0.07

**Table 6:** Elongation at break and elongation at break/section relation values per specimen and medium values.

Specimen	Elongation	Elongation/Section
	(%)	(%/mm^2^)
3	14.32	0.18
8	12.80	0.16
12	11.50	0.14
Medium value a (3, 8, 12)	12.87	0.16
Standard deviation	1.41	0.02
1C	11.18	0.14
2C	13.06	0.16
4C	8.18	0.10
Medium value b (1C, 2C, 4C)	10.81	0.13
Standard deviation	2.46	0.03
5C	10.04	0.13
6C	9.72	0.12
7C	10.40	0.13
Medium value c (5C, 6C, 7C)	10.05	0.12
Standard deviation	0.34	0.01
9C	10.74	0.13
10C	12.76	0.15
11C	11.12	0.14
Medium value d (9C, 10C, 11C)	11.54	0.14
Standard deviation	1.07	0.01
Corrosion total medium value	10.80	0.13
(MVt = (MVb + MVc + MVd)/3)		
Standard deviation	0.75	0.01

**Table 7:** Tensile strength/elongation relation values per specimen and medium values.

Specimen	Tensile Strength/Elongation (MPa/%)
3	38.96
8	42.77
12	48.40
Medium value a (3, 8, 12)	43.38
Standard deviation	4.75
1C	49.89
2C	42.45
4C	67.03
Medium value b (1C, 2C, 4C)	53.12
Standard deviation	12.60
5C	55.12
6C	56.97
7C	52.57
Medium value c (5C, 6C, 7C)	54.88
Standard deviation	2.21
9C	50.47
10C	43.45
11C	48.27
Medium value d (9C, 10C, 11C)	47.40
Standard deviation	3.59
Corrosion total medium value	51.80
(MVt = (MVb + MVc + MVd)/3)	
Standard deviation	3.91

**Table 8:** Yield strength/section values per specimen and medium values vs. batch.

Batch	Yield Strength/Section Specimen without Corrosion (1) (MPa/mm^2^)	Yield Strength/Section Medium Values with Corrosion (2) (MPa/mm^2^)	(1)*/*(2) (%)
1	6.25 (specimen 3)	6.09 (MVb)	2.59
2	6.05 (specimen 8)	6.04 (MVc)	0.13
3	6.08 (specimen 12)	5.89 (MVd)	3.33
Extreme values variation (%)	3.21	3.43	

**Table 9:** Tensile strength/Section values per specimen and Medium values vs. Batch.

Batch	Tensile Strength/Section Specimen without Corrosion (1) (MPa/mm^2^)	Tensile Strength/Section Medium Values with Corrosion (2) (MPa/mm^2^)	(1)*/*(2) (%)
1	7.00 (specimen 3)	6.85 (MVb)	2.17
2	6.81 (specimen 8)	6.85 (MVc)	−0.48
3	6.94 (specimen 12)	6.63 (MVd)	4.69
Extreme values variation (%)	2.73	3.41	

**Table 10:** Elongation/Section values per specimen and Medium values vs. Batch.

Batch	Elongation/Section Specimen without Corrosion (1) (%/mm^2^)	Elongation/Section Medium Values with Corrosion (2) (%/mm^2^)	(1)*/*(*2*) (%)
1	0.18 (specimen 3)	0.13 MVb	33.97
2	0.16 (specimen 8)	0.12 MVc	27.60
3	0.14 (specimen 12)	0.14 MVd	2.06
Extreme values variation (%)	25.38	12.46	
